# Clinical diagnoses of patients showing caloric inversion

**DOI:** 10.1016/j.bjorl.2023.101378

**Published:** 2023-12-15

**Authors:** Hansol Kim, Haeun Souh, Dong-Han Lee, Jung Eun Shin, Chang-Hee Kim

**Affiliations:** Konkuk University Medical Center, Research Institute of Medical Science, Konkuk University School of Medicine, Department of Otorhinolaryngology-Head and Neck Surgery, Seoul, Republic of Korea

**Keywords:** Bithermal caloric test, Dizziness, Caloric inversion, Paradoxical caloric response, Chronic otitis media

## Abstract

**Objectives:**

When air irrigation is used for caloric stimulation in patients with a perforated ear, warm irrigation may elicit a nystagmus that initially beats in the opposite direction of what is expected for warm irrigations, which is referred to as “caloric inversion”. This study aimed to investigate the disease group in which caloric inversion appeared in patients who underwent caloric testing and to classify the patterns of caloric inversion.

**Methods:**

We conducted a retrospective review of bithermal caloric test results that were collected in our dizziness clinic between 2005 and 2022. Caloric inversion was defined when nystagmus induced by caloric stimulation appeared in the opposite direction to that expected. The incidence of caloric inversion among all patients who underwent bithermal caloric tests was calculated. To confirm the clinical diagnoses of the patients with caloric inversion, their clinical records were reviewed.

**Results:**

Out of 9923 patients who underwent bithermal caloric tests, 29 patients (0.29%) showed a caloric inversion. The most common clinical diagnosis was chronic otitis media (21 of 29, 72%). Of the 21 patients with chronic otitis media, 20 patients showed a caloric inversion by warm air irrigation and one patient showed caloric inversion by cold air stimulation. Patients with clinical diagnoses other than chronic otitis media such as sudden sensorineural hearing loss, benign paroxysmal vertigo of childhood and recurrent vestibulopathy showed caloric inversion by warm air irrigation. Caloric inversion by warm water irrigation was observed in patients with lateral semicircular canal cupulopathy and recurrent vestibulopathy. Two patients (one with Meniere’s disease and one with age-related dizziness) showed caloric inversion by cold water irrigation.

**Conclusion:**

Caloric inversion can be observed in various diseases other than chronic otitis media with tympanic membrane perforation. Special care should be taken in the interpretation of caloric test results.

**Level of Evidence:**

Level 4.

## Highlights

• Caloric inversion was observed in 0.29% of patients who underwent caloric tests.
• Caloric inversion can be observed in various diseases without TM perforation.
• Clinicians should be careful in interpreting caloric tests in dizzy patients.

## Introduction

A bithermal caloric test, in which the external auditory meatus is irrigated by a medium with significantly different temperature from the body temperature, is one of the most practical vestibular function tests. Despite the limitation that caloric stimulation is not a physiologic stimulation, the bithermal caloric test offers a major advantage over other vestibular tests in that caloric irrigation allows each labyrinth to be investigated independently. The bithermal caloric test still suffers from several shortcomings. For example, caloric irrigation in patients with tympanic membrane perforation has several issues to be considered. Open-loop water irrigation cannot be used in the perforated ear due to the concern of excessive stimulation or infection, and a quantitative comparison of caloric responses from both ears may not be possible because the crucial assumption that both ears receive equal stimulation is not valid in the case of a perforated ear. When air irrigation is used for caloric stimulation in patients with a perforated ear, warm irrigation may elicit a nystagmus that initially beats in the opposite direction of what is expected for warm irrigations.^1^

The term “caloric inversion” refers to the phenomenon where the nystagmus induced by caloric stimulation appears in the opposite direction to that expected.^1^ In other words, the fast phase of nystagmus moves toward the unstimulated ear when warm air or water flows into the ear and moves toward the stimulated ear when cold air or water flows into the ear. The presence of caloric inversion by warm air irrigation has been demonstrated in chronic otitis media patients with tympanic membrane perforation many times^2^, ^3^, ^4^, ^5^, ^6^, ^7^, ^8^ since the first report.^1^ This phenomenon is believed to occur due to the evaporative cooling effect that dry warm air irrigation has on the moist mucous membrane in the middle ear cavity.

Although varying patterns of caloric inversion, such as disconjugate nystagmus elicited by cold air irrigation in patients with tympanic membrane perforation, were demonstrated in the first report,^1^ subsequent investigations have focused on caloric inversion by warm air irrigation in patients with tympanic membrane perforation.^2^, ^3^, ^4^, ^5^, ^6^, ^7^, ^8^

Therefore, this study aimed to investigate the disease group in which caloric inversion appeared in patients who underwent caloric testing and to classify the patterns of caloric inversion.

## Methods

This study was conducted on 9923 patients who underwent bithermal caloric tests at our vestibular function test laboratory from August 2005 to March 2022. All data were retrospectively collected through an electronic medical record system and included the patient’s sex, age, diagnosis and lesion location and the results of vestibular function tests such as the spontaneous nystagmus test and caloric nystagmus test.

A bithermal caloric test was performed by irrigating 30 °C and 44 °C water into both ears for 40 s while the patient was lying supine with the head tilted 30° forward from the horizontal plane. When there was a risk of infection or damage due to water-based testing in cases of chronic otitis media after surgery or chronic otitis media with tympanic membrane perforation, air irrigation was conducted.^1^, ^4^, ^5^, ^8^ The patients who were not able to tolerate the water-based caloric test, such as vestibular neuritis patients with severe vertigo and nausea and pediatric patients, underwent air caloric irrigation even though they had no tympanic membrane perforation. The air-based caloric test involved irrigating 24 °C and 50 °C air into both ears for 60 s. Eye movements were recorded using a video nystagmography device (CHARTR VNG, ICS medical, Schaumburg, USA), and peak Slow-Phase Velocity (SPV) was calculated for each ear after cold/warm water/air irrigation. The scanning frequency and image resolution of a video nystagmography device was 60 Hz and 320 (horizontal) X 240 (vertical) pixels, respectively. After subtracting the horizontal component of the spontaneous nystagmus from the peak SPV values of caloric nystagmus, we examined whether the corrected peak SPV values came out in the opposite direction to what was predicted. Then, a caloric pod was evaluated if the plot of the caloric pod demonstrated caloric inversion as the corrected peak SPV values. The caloric inversion was determined to be positive if a caloric inversion was observed in at least one of the four stimulations (warm and cold in both ears). To confirm the clinical diagnoses of the patients with caloric inversion, their clinical records, including past medical history, neurotological examinations, audiometric and vestibular evaluations, and imaging results, were reviewed.

## Results

**Figure 1:**
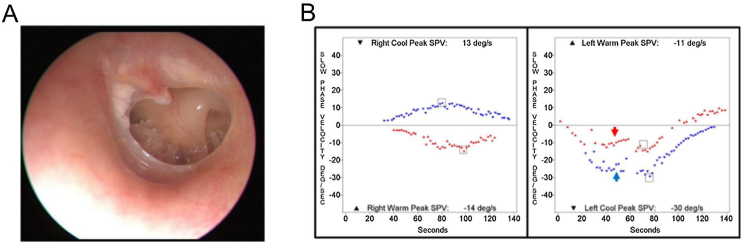
Representative case of caloric inversion in chronic otitis media with tympanic membrane perforation. (A) An otoendoscopic examination demonstrated a tympanic membrane perforation on the left side. (B) A caloric inversion by warm air irrigation was observed on the left side (red arrow). Note that hyperactive responses to cold air irrigation on the affected side were observed (blue arrow).

**Figure 2:**
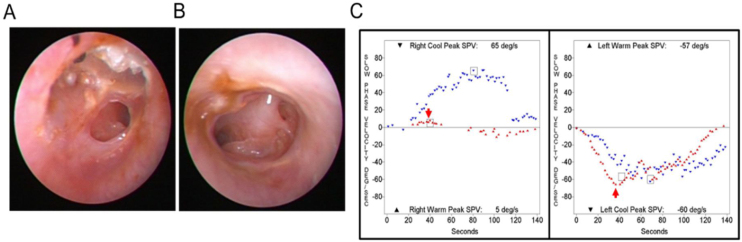
A 77-year-old patient with bilateral chronic otitis media. Otoendoscopic examination showed tympanic membrane perforation on the right (A) and left side (B). (C) A caloric inversion by warm air irrigation was observed on both sides (red arrows).

**Figure 3:**
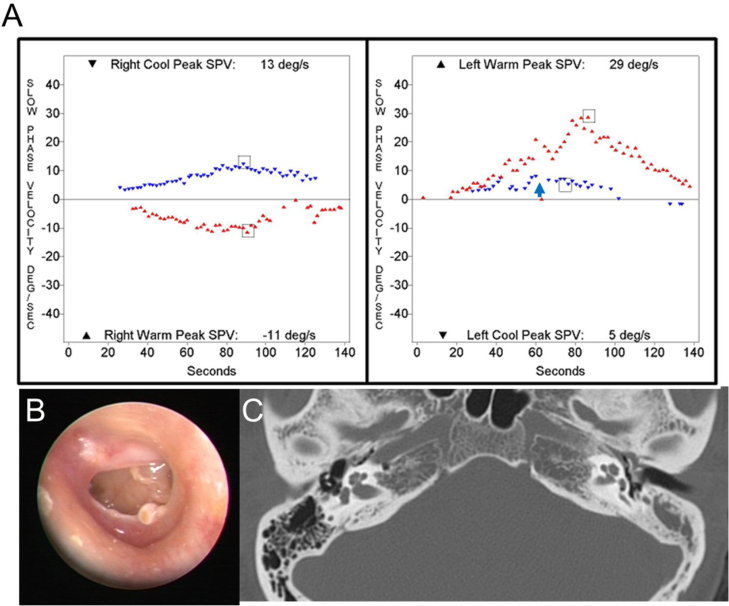
A 49-year-old patient with left chronic otitis media. (A) A caloric inversion by cold air irrigation was observed on the affected side (blue arrow). (B) Otoendoscopic examination showed tympanic membrane perforation on the left side. (C) Temporal bone computed tomography showed tympanosclerosis and a sclerotic mastoid cavity filled with a soft tissue density on the left side.

Out of 9923 patients who underwent bithermal caloric tests, 29 patients (0.29%) showed a caloric inversion. The clinical diagnoses of patients showing caloric inversion are listed in Table 1, and clinical diagnosis of patients with caloric inversion according to application methods for caloric stimulation is presented in Table 2. Otoscopic findings and SPV of caloric response and spontaneous nystagmus were described in Table 3. The most common clinical diagnosis was chronic otitis media (21 of 29, 72%). Of the 21 patients with chronic otitis media, 19 patients had unilateral tympanic membrane perforation and showed caloric inversion only when warm air irrigation was performed on the affected ear (Fig. 1). One patient had bilateral chronic otitis media with tympanic membrane perforation and showed a caloric inversion by warm air irrigation on both sides (nº 10 in Table 3; Fig. 2). Another patient with left chronic otitis media with tympanic membrane perforation, interestingly, showed caloric inversion only when cold air was irrigated in the affected ear (nº 14 in Table 3; Fig. 3A). This patient visited our clinic with a chief complaint of acute vertigo that was aggravated by head movement and reported known tympanic membrane perforation on the left side since childhood (Fig. 3B). Weak geotropic positional nystagmus was observed in the supine head roll test, and neurologic examination revealed no focal neurologic deficit. Temporal bone computed tomography showed the sclerotic mastoid cavity filled with soft tissue density and tympanosclerosis on the left side (Fig. 3C), and brain magnetic resonance imaging revealed left chronic otomastoiditis and a 3 mm meningioma in the left prepontine-cerebellopontine cistern along the anterior tentorial margin, without abnormal findings in the brain parenchyma, cranial nerves or inner ear (Table 2).

**Table 1:** Diagnoses of patients with caloric inversion (n = 29).

Diagnosis	Number of patients (%)
Chronic otitis media	21 (72%)
Lateral semicircular canal cupulopathy	2 (7%)
Sudden sensorineural hearing loss	1 (3%)
Meniere’s disease	1 (3%)
Benign paroxysmal vertigo of childhood	1 (3%)
Age-related dizziness	1 (3%)
Recurrent vestibulopathy	2 (7%)

**Table 2:** Clinical diagnoses of patients with caloric inversion according to the application methods of caloric stimulation (n = 29).

	Warm irrigation	Cold irrigation
Air	Chronic otitis media (n = 20)	Chronic otitis media (n = 1)
	Recurrent vestibulopathy (n = 1)	
	Benign paroxysmal vertigo of childhood (n = 1)	
	Sudden sensorineural hearing loss (n = 1)	

Water	Lateral semicircular canal cupulopathy (n = 2)	Meniere’s disease (n = 1)
	Recurrent vestibulopathy (n = 1)	Age-related dizziness (n = 1)

**Table 3:** Tympanic membrane appearance and caloric response in patients with caloric inversion (n = 29).

Nº	Sex	Age	Diagnosis	Side	TM appearance	Maximal slow phase velocity (°/s)				
						SN	RW	RC	LW	LC
1	M	52	Chronic otitis media	R	Central moderate perforation	0	18	45	5	−7
2	M	66	Chronic otitis media	R	Central small perforation	0	2	15	15	−15
3	M	79	Chronic otitis media	R	Central large perforation	−1	11	24	11	−10
4	M	20	Chronic otitis media	R	Central large perforation	0	5	38	10	−27
5	F	75	Chronic otitis media	L	Central large perforation	−2	−5	6	−3	−6
6	M	44	Chronic otitis media	R	Central moderate perforation	−1	10	49	6	−7
7	F	74	Chronic otitis media	L	Near total perforation	0	−18	19	−17	−22
8	M	74	Chronic otitis media	R	Adhesion	0	25	50	34	−17
9	F	78	Chronic otitis media	L	Anterior moderate perforation	0	−25	17	−4	−14
10	M	77	Chronic otitis media	R	Epitympanic cholesteatoma	−1	5	65	−57	−60
			Chronic otitis media	L	Central large perforation					
11	M	74	Chronic otitis media	R	Central large perforation	1	3	10	11	−7
12	F	70	Chronic otitis media	R	Near total perforation	−2	20	66	60	−37
13	F	51	Chronic otitis media	L	Near total perforation	0	−24	25	−63	−82
14	F	49	Chronic otitis media	L	Central large perforation	1	−11	13	29	5
15	M	67	Chronic otitis media	L	Central moderate perforation	0	−8	23	−6	−34
16	F	64	Chronic otitis media	L	Central large perforation	−2	−11	7	−9	−20
17	F	42	Chronic otitis media	L	Central large perforation	0	−14	13	−11	−30
18	F	48	Chronic otitis media	R	Central large perforation	1	12	32	14	−18
19	F	64	Chronic otitis media	L	Inferior small perforation	0	−5	16	−2	−20
20	M	65	Chronic otitis media	R	Central large perforation	0	9	26	9	−6
21	F	48	Chronic otitis media	R	Central small perforation	0	18	31	2	−3
22	F	61	Meniere’s disease	R	Normal	23	15	8	46	−8
23	M	72	Recurrent vestibulopathy	N/A	Normal	5	6	8	8	−8
24	M	55	Lateral semicircular canal light cupula	L	Normal	−1	−11	10	−2	−13
25	F	11	Benign paroxysmal vertigo of childhood	N/A	Normal	2	19	28	10	−12
26	F	80	Age-related dizziness	N/A	Normal	1	−30	−8	31	−14
27	F	54	Recurrent vestibulopathy	N/A	Normal	2	6	13	−4	−9
28	F	73	Lateral semicircular canal cupulolithiasis	L	Normal	−1	−8	12	−6	−3
29	F	69	Sudden sensorineural hearing loss with vertigo	R	Normal	0	4	5	13	−11

R, Right; L, Left; TM, Tympanic Membrane; SN, Spontaneous Nystagmus; RW, Right Warm; RC, Right Cold; LW, Left Warm; LC, Left Cold; N/A, Not Applicable.

**Figure 4:**
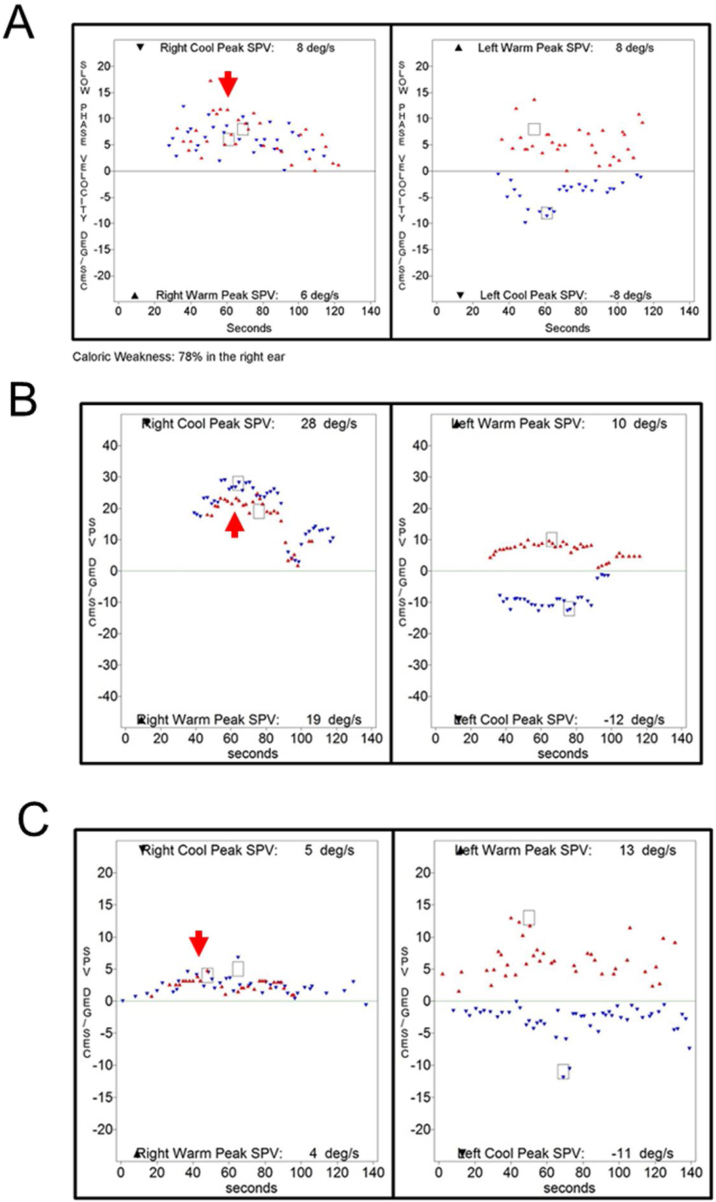
Caloric inversion by warm air irrigation in patients without chronic otitis media. (A) A 72-year-old patient with recurrent vestibulopathy showed caloric inversion by warm air irrigation on the right side (red arrow). (B) An 11-year-old patient with benign paroxysmal vertigo of childhood showed caloric inversion by warm air irrigation on the right side (red arrow). (C) A 69-year-old patient with sudden sensorineural hearing loss on the right side showed caloric inversion by warm air irrigation on the right side (red arrow).

Caloric inversion by warm air irrigation was observed in conditions other than chronic otitis media (Table 2). A 72-year-old man visited our emergency department with acute spontaneous vertigo and severe nausea (nº 23 in Table 3). This episode was his third attack of vertigo, which was not accompanied by hearing loss or other neurologic symptoms. The patient showed left-beating spontaneous nystagmus (SPV = 5°/s), which was augmented by horizontal headshaking (SPV = 25°/s). A bedside head impulse test revealed corrective catch-up saccade on the right side, and other neurologic examinations revealed no abnormalities. An otoendoscopic examination showed a normal tympanic membrane on both sides, and pure tone audiometry showed symmetric age-related hearing loss in both ears. Brain MRI and MRA demonstrated no abnormal findings except severe stenosis in the right vertebral artery V4 segment. This patient was diagnosed with recurrent vestibulopathy.^9^, ^10^ Because the patient could not tolerate water irrigation due to severe nausea, a bithermal caloric test was conducted with air irrigation, which showed caloric inversion by warm air irrigation on the right side (Fig. 4A). An 11-year-old girl who was diagnosed with benign paroxysmal vertigo of childhood showed caloric inversion by warm air irrigation on the right side (nº 25 in Table 3; Fig. 4B). A 69-year-old woman had been diagnosed with sudden sensorineural hearing loss with vertigo on the right side. Because a caloric test was performed after intratympanic steroid injection, air irrigation was used for caloric stimulation, which showed caloric inversion by warm air irrigation on the right side (nº 29 in Table 3; Fig. 4C).

**Figure 5:**
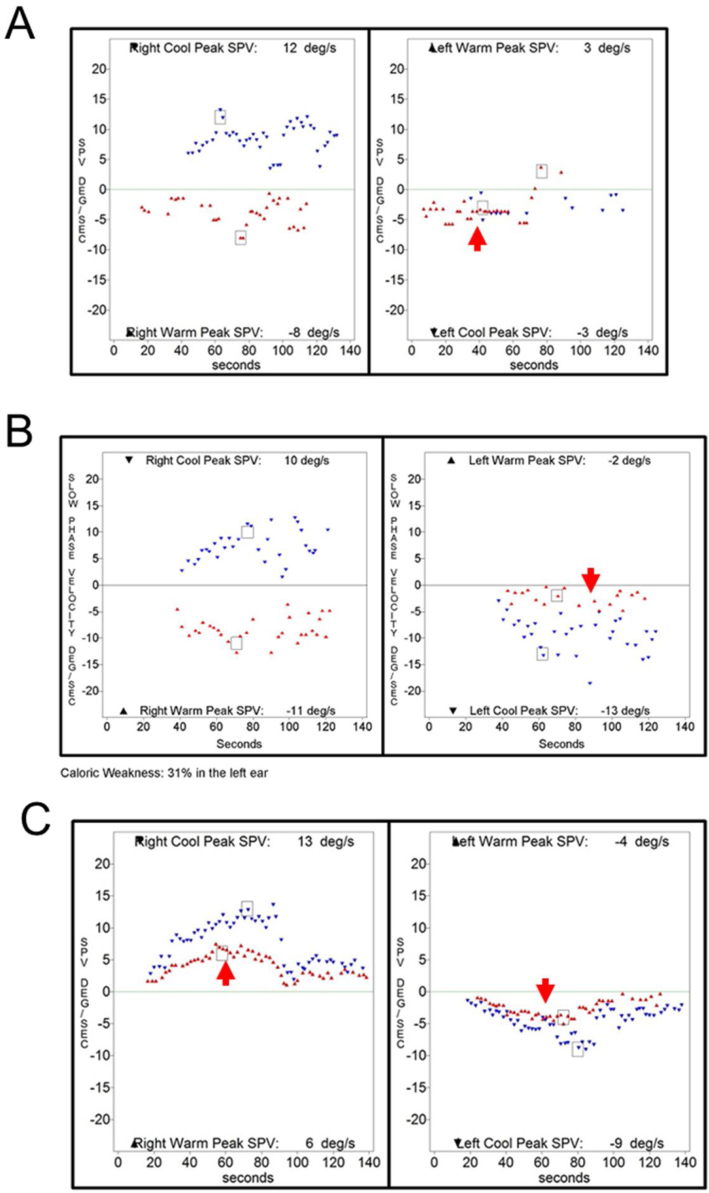
Caloric inversion by warm water irrigation in the ear with normal tympanic membrane. (A) A 73-year-old patient with left lateral semicircular canal cupulolithiasis showed caloric inversion by warm water irrigation on the left side (red arrow). (B) A 55-year-old patient with a left lateral semicircular canal light cupula showed caloric inversion by warm water irrigation on the left side (red arrow). (C) A 54-year-old patient with recurrent vestibulopathy showed caloric inversion by warm water irrigation on both sides (red arrows).

Interestingly, the present study showed that a caloric inversion was observed with caloric stimulation by water irrigation in the intact tympanic membrane. Caloric inversion by warm water irrigation was observed in 2 patients with lateral semicircular canal cupulopathy (Table 2), one with cupulolithiasis on the left side (nº 28 in Table 3; Fig. 5A)^11^ and the other with light cupula on the left side (nº 24 in Table 3; Fig. 5B).^12^ Neurologic examination and brain MRI revealed no abnormal findings in either patient. Another patient who was diagnosed with recurrent vestibulopathy showed caloric inversion by warm water irrigation on both sides (nº 27 in Table 3; Fig. 5C). An otoscopic examination revealed a normal tympanic membrane, and neurological examination was nonspecific. No abnormal findings were observed on TBCT or brain MRI.

**Figure 6:**
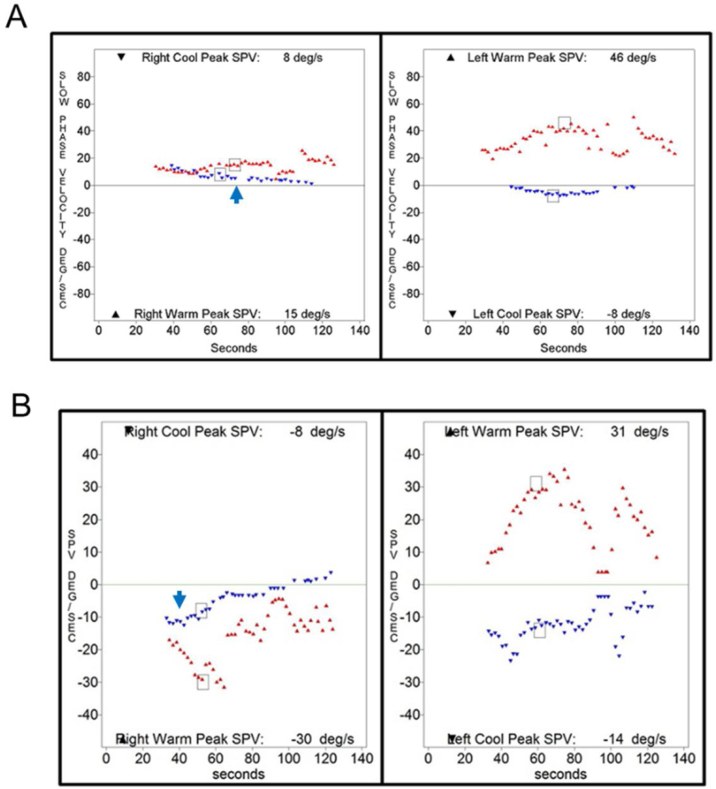
Caloric inversion by cold water irrigation in an ear with a normal tympanic membrane. (A) A 61-year-old patient with definite Meniere’s disease on the right side showed caloric inversion by cold water irrigation in the right ear (blue arrow). (B) An 80-year-old patient with chronic dizziness showed caloric inversion by cold water irrigation in the right ear (blue arrow).

Two patients with normal tympanic membranes showed caloric inversion by cold water irrigation (Table 2). A 61-year-old patient who met the diagnostic criteria of Meniere’s disease^13^, ^14^ on the right side showed caloric inversion by cold water irrigation (nº 22 in Table 3; Fig. 6A). Spontaneous nystagmus beating toward the left side (SPV = 23°/s) was observed, and no abnormal findings were observed on TBCT or temporal bone MRI. This patient underwent two more caloric tests during another episode of acute vertigo, which showed no caloric inversion (Supplemental Fig. 1 A and B). An 80-year-old patient with age-related dizziness showed caloric inversion by cold water irrigation (nº 26 in Table 3; Fig. 6B). The patient complained of chronic dizziness, and had the comorbidities of anemia, hypertension, and arrhythmia. Neurologic examination was nonspecific, but brain MRI and MRA showed diffuse brain atrophy. A bithermal caloric test was conducted again 19-months after the first one, which showed no caloric inversion (Supplemental Fig. 1C).

## Discussion

The present study demonstrated that caloric inversion can be observed in not only chronic otitis media with tympanic membrane perforation but also, extremely rarely, other disease entities with normal tympanic membranes such as recurrent vestibulopathy, Meniere’s disease, benign paroxysmal vertigo of childhood and lateral semicircular canal cupulopathy. Furthermore, caloric inversion can be elicited by not only warm air irrigation but also cold air, cold water, or warm water irrigation.

In previous studies, caloric inversion by dry warm air irrigation has been reported in chronic otitis media with tympanic membrane perforation,^1^, ^2^, ^3^, ^4^, ^5^, ^6^, ^7^ and an evaporative cooling effect on the moist mucous lining of the middle ear cavity was suggested as a possible mechanism. Barber et al. supported this mechanism by showing that caloric inversion was not observed when the warm air was saturated with water vapor.^1^ This idea was further supported by an experiment using physical model components composed of PVC pipe mimicking the external auditory canal, a hood, a thermometer, and a caloric air stimulator, which showed that the temperature of a moist hood was actually decreased by dry, warm air stimulation.^2^ Caloric inversion occurs in 37%–40% of patients with tympanic membrane perforation, and the occurrence of caloric inversion is not correlated with the perforation size.^2^, ^3^

In the present study, we sought to investigate the incidence and clinical diagnoses of patients showing caloric inversion. Caloric inversion was observed in only 0.29% of our patients who underwent a bithermal caloric test. Chronic otitis media with tympanic membrane perforation was the most common clinical diagnosis causing caloric inversion, which was consistent with previous observations.^1^, ^2^, ^3^, ^4^, ^5^, ^6^, ^7^ While most (20 of 21) caloric inversion was induced by warm air stimulation in the perforated ear, caloric inversion by cold air irrigation was observed in one case. This finding could not be explained by evaporative cooling of the mucus lining the middle ear cavity. Barber et al. reported that, in a patient with large perforation of the right tympanic membrane, disconjugate caloric nystagmus was observed when cold air stimulation was applied to the affected ear, in the form of right-beating nystagmus in the right eye and left-beating nystagmus in the left eye.^1^ The patient had no evidence of Central Nervous System (CNS) disease, and conventional conjugate left-beating nystagmus was elicited when they repeated the cold caloric stimulation with cold tap water after introducing a rubber finger cot into the depths of the external auditory canal.^1^ Although they took this finding as proof of peripherally caused disconjugate eye movement, the mechanism underlying this was not clearly elucidated. In addition, interestingly, caloric inversion was observed by cold water irrigation in one patient with Meniere’s disease and age-related dizziness. Nagle reported that 4 °C ice water stimulation may produce disconjugate caloric nystagmus with a fast component in each eye, toward the nose, in healthy human subjects.^15^ In animal studies using normal cats and rabbits, caloric inversion can be elicited by repeated cold caloric irrigations.^16^, ^17^, ^18^

We observed caloric inversion by warm irrigation in diseases other than chronic otitis media with tympanic membrane perforation. A patient with sudden sensorineural hearing loss underwent a bithermal caloric test using air irrigation because the patient had received intratympanic steroid injection prior to a caloric test. We assume that evaporative cooling from the steroid solution within the external auditory canal might have produced caloric inversion in the affected ear. In one patient with benign paroxysmal vertigo of childhood and one with recurrent vestibulopathy, warm air irrigation produced unilateral caloric inversion, of which the underlying mechanism remains uncertain. Warm water irrigation elicited caloric inversion in two patients with lateral semicircular canal cupulopathy. Regardless of the mechanism, the finding that the side of caloric inversion corresponded to that of cupulopathy may support a peripheral cause rather than a CNS effect underlying the caloric inversion. Bilateral caloric inversion by warm water irrigation was observed in one patient with recurrent vestibulopathy, the mechanism of which requires further investigation.

The present study demonstrated that caloric can be observed in various clinical conditions other than chronic otitis media such as Meniere’s disease, lateral semicircular canal cupulopathy, sudden sensorineural hearing loss, benign paroxysmal vertigo of childhood, age-related dizziness and recurrent vestibulopathy. In addition, caloric inversion can be elicited by various application methods for caloric stimulation other than warm air irrigation such as cold air irrigation in chronic otitis media, cold water irrigation in Meniere’s disease and age-related dizziness, and warm water irrigation in lateral semicircular canal cupulopathy. Thus, to avoid misinterpretation of caloric responses, it is recommended that each maximal SPV value of binaural and bithermal caloric stimulation after subtraction of spontaneous nystagmus should be checked in all patients who conducted caloric tests.

## Conclusion

Lessons from the present study are as follows: (1) Because caloric inversion, although extremely rarely, can be observed in various diseases without tympanic membrane perforation, clinicians are recommended to check the shape of caloric pod plots when evaluating the results of vestibular function tests. (2) Clinicians should be careful in interpreting the results of a caloric test for the evaluation of dizziness in patients with tympanic membrane perforation because the caloric response can be biased. Walther et al. suggested near-infrared radiation as a reliable alternative method of warm air caloric stimulation.^19^

## Conflicts of interest

The authors declare no conflicts of interest.
