# Supplementary material for: Clinical diagnoses of patients showing caloric inversion
Source: Braz J Otorhinolaryngol. 2023 Dec 15;90(2):101378. doi: 10.1016/j.bjorl.2023.101378 (PMC10826153; doi:10.1016/j.bjorl.2023.101378)

BJORL-D-23-00256_Supplementary Material

**Supplemental Figure 1** The patient with Meniere’s disease underwent two more caloric tests during another episode of acute vertigo, which showed no caloric inversion (A and B). (C) A bithermal caloric test was performed again 19 months after the first caloric test in a patient with age-related dizziness, and caloric inversion was not observed.


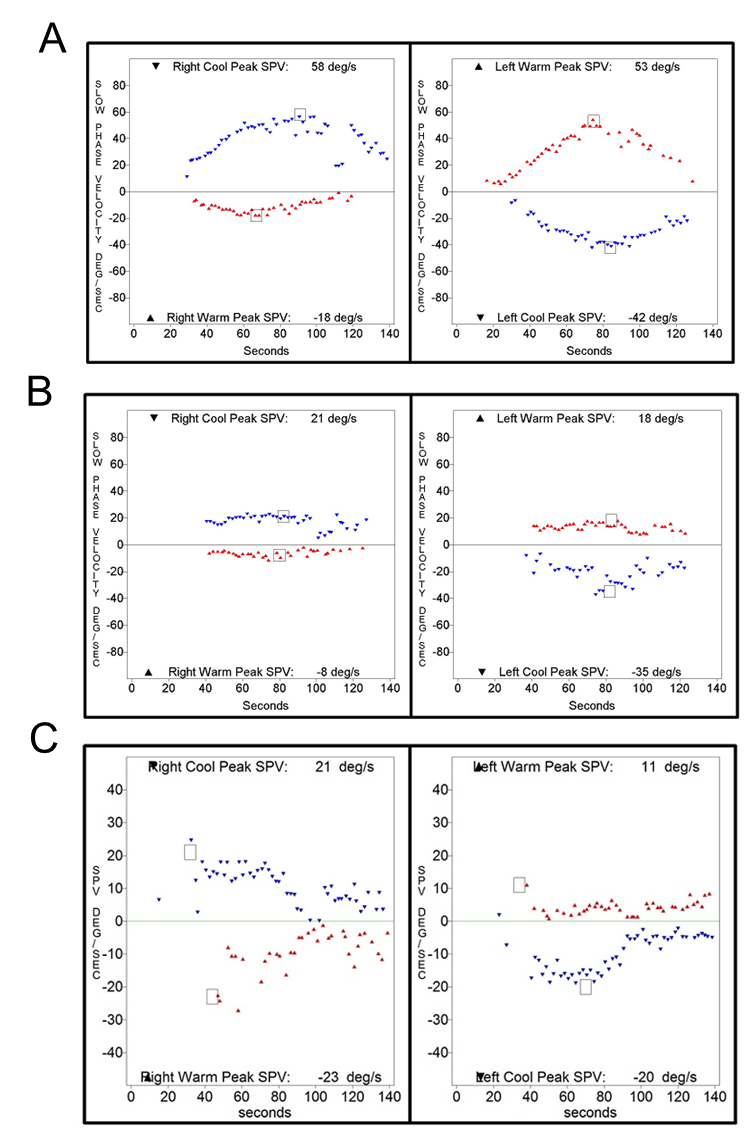

Supplement: Supplementary file 1 [file mmc1.docx]
